# Development and Characterization of Emulsion-Templated Oleogels from Whey Protein and Spent Coffee Grounds Oil

**DOI:** 10.3390/foods14152697

**Published:** 2025-07-31

**Authors:** Aikaterini Papadaki, Ioanna Mandala, Nikolaos Kopsahelis

**Affiliations:** 1 Department of Food Science and Technology, Ionian University, 28100 Argostoli, Greece; kpapadaki@ionio.gr; 2 Department of Food Science and Human Nutrition, Agricultural University of Athens, Iera Odos 75, 11855 Athens, Greece; imandala@aua.gr

**Keywords:** coffee by-products, bacterial nanocellulose, fat substitutes, protein oleogels, gel structure, rheology, sustainable foods

## Abstract

This study aimed to develop novel oleogels using whey protein (WP) and bacterial cellulose nanowhiskers (BCNW) to expand the potential applications of spent coffee grounds oil (SCGO). An emulsion-templated approach was employed to structure SCGO with varying WP:SCGO ratios, while the incorporation of BCNW was evaluated as a potential stabilizing and reinforcing agent. All oleogels behaved as “true” gels (tan δ < 0.1). Rheological analysis revealed that higher WP content significantly increased gel strength, indicating enhanced structural integrity and deformation resistance. The addition of BCNW had a significant reinforcing effect in oleogels with a higher oil content (WP:SCGO 1:5), while its influence was less evident in formulations with lower oil content (WP:SCGO 1:2.5). Notably, depending on the WP:SCGO ratio, the storage modulus (G′) data showed that the oleogels resembled both hard (WP:SCGO 1:2.5) and soft (WP:SCGO 1:5) solid fats, highlighting their potential as fat replacers in a wide range of food applications. Consequently, this study presents a sustainable approach to structuring SCGO while tailoring its rheological behavior, aligning with global efforts to reduce food waste and develop sustainable food products.

## 1. Introduction

Conventional fat products widely used in the food industry include palm oil, hydrogenated fats and partially hydrogenated fats. From a technological point of view, these fats contribute to the structural properties of many food products by providing a semi-solid consistency, making them highly desirable in food formulations. However, growing public awareness has raised concerns, as excessive consumption of saturated and *trans* fatty acids has been correlated with increased risk of diet-related diseases, such as obesity and cardiovascular disease [1]. Consequently, there is a growing interest in replacing these fats with structured oils or oleogels, rich in unsaturated fatty acids, to support healthier dietary choices [2]. However, further research is needed before commercialization to ensure that oleogels replicate the technological and sensory characteristics of conventional fats and ultimately gain high consumer acceptance [3].

The oil-structuring potential of proteins has gained increasing attention for the development of safe, clean-label oleogels with significant potential to replace traditional solid fats in foods [4]. The unique properties of protein-based oleogels, such as their ability to mimic the texture and functionality of solid fats, make them ideal candidates for use in various food applications. Preparation methods such as emulsion-templated and direct dispersion of dried protein aggregates, have a significant effect on their properties, thus tailoring them for specific applications such as spreads, meat and bakery products, offering a promising alternative to traditional fats [5]. The formulation of emulsion-templated oleogels includes the incorporation of an oil phase into a protein hydrogel network, followed by water removal through evaporation. In these systems, the oil is not inherently structured but is instead stabilized within the protein matrix. In many cases, polysaccharides, such as flaxseed gum, xanthan gum, κ-carrageenan, alginate and low methoxyl pectin, have been studied in combination with proteins, primarily to enhance the stability and reinforce the structure of oleogel, emulgel or emulsion systems [5,6].

Furthermore, proteins and polysaccharides have the advantage of being derived not only from animal- or plant-based materials but also from renewable resources, thereby supporting the growing demand for healthier and more sustainable food products. Likewise, the importance of producing food products from renewable resources is becoming increasingly recognized. This approach aligns with environmental sustainability and resource efficiency goals, such as those outlined in the European Green Deal and the United Nations 17 Sustainable Development Goals. Currently, several renewable resources, such as microbial oils and marine by-products, have been employed in the development of novel food products, including oleogels [7,8,9]. In this context, increased attention has been given to the valorization of spent coffee ground (SCG), a by-product generated during the coffee brewing process. The abundance of compounds present in SCG makes it a promising ingredient for enhancing the nutritional value of food products. For example, SCG have been utilized in baked goods and meat products to improve their functional and nutritional properties [10,11]. SCG have also been utilized in soap production, mixed with other renewable resources [12], or incorporated into electrospun composites for structuring castor oil as sustainable alternative to lubricants [13]. Additionally, SCG oil (SCGO) has been utilized in cosmetics formulations [14], whereas our research group has also proposed its use for the enzymatic synthesis of wax esters with oil-structuring properties [15]. Building on this strategy to valorize renewable resources, bacterial cellulose, especially in the form of nanowhiskers, nanocrystals or nanofibrils, has attracted significant attention for its ability to structure and stabilize emulsions and edible films [16,17,18,19,20]. However, its application in oleogels remains limited.

In light of the aforementioned, this study aimed to develop novel oleogel formulations using whey protein and bacterial cellulose, with the ultimate goal of expanding the potential applications of SCGO. Specifically, the emulsion-templated approach was applied and the research focused on structuring SCGO with varying contents of whey protein. Furthermore, this study is the first to investigate the impact of bacterial cellulose nanowhiskers (BCNW) incorporation on the rheological properties of oleogels and the hypothesis that incorporating BCNW into oleogels would enhance their stability.

## 2. Materials and Methods

### 2.1. Raw Materials and Reagents

Whey protein concentrate (WP), with a protein content of ~80% *w*/*w*, was purchased by Hellenic Proteins (WHEYPRO80, Athens, Greece). SCG were collected from a local coffee shop (Arabica variety, Argostoli, Kefalonia, Greece) and subsequently oven-dried. SCGO was obtained through solvent extraction (SCG:hexane at a ratio of 1:9) according to the protocol described in our previous study [15]. Bacterial cellulose production, purification and hydrolysis to obtain BCNW, were performed according to our previous protocol [16]. All reagents used were of analytical grade.

### 2.2. Preparation of Emulsion-Templated Oleogels

An emulsion-templated approach was employed for the development of whey protein oleogels. Initially, a WP hydrogel was produced following the protocol of Plazzota et al. [21]. Briefly, an aqueous WP solution (20%, *w*/*w*) was prepared, stirred continuously for 2 h and then stored at 4 °C overnight. Following pH adjustment to 5.7, the solution was heated to 85 °C for 15 min, and then rapidly cooled down to room temperature. Then, the resulting WP hydrogel was mixed with SCGO and homogenized at 13,000 rpm for 3 min (HG-15D, Witeg Labortechnik GMBH, Wertheim, Germany). Subsequently, the emulsions were placed at 80 °C for 24 h and then lyophilized (BK-FD12P, Biobase Biodustry Co., Ltd., Jinan, China). The lyophilized oleogels were then manually processed using a spatula for 5 min until a homogeneous mixture was produced [22]. Oleogels with varying ratios of WP to SCGO were prepared (1:2.5 and 1:5), and the impact of 5% (*w*/*w* of WP weight) BCNW addition (just before the pH adjustment step) on the properties of the oleogels was assessed. The different oleogels were labeled as WP:SCGO 1:2.5, WP:SCGO 1:2.5 BCNW, WP:SCGO 1:5 and WP:SCGO 1:5 BCNW.

### 2.3. Characterization of Oleogels

#### 2.3.1. Color

The color characteristics of the oleogels were determined (ChromaMeter CR-400/410, Konica Minolta, Tokyo, Japan) and color data were collected in the CIE-L*a*b* color space. Before the color measurement the colorimeter was calibrated with a standard white and black plates and L*, a*, b* values were determined. In addition, color characteristics, such as chroma (C*) and hue angle (h°) were also determined [7].

#### 2.3.2. Rheological Analysis

The Discovery HR3 hybrid Rheometer was utilized to determine the rheological characteristics (TA Instruments, New Castle, DE, USA). The analysis was performed using a gap of 700 μm and a plate to plate geometry (40 mm diameter) [15]. The oleogels were kept at room temperature, gently loaded onto the Peltier plate using a plastic spatula and allowed to rest for 120 s prior to starting the experiment, ensuring thermal and structural equilibration. Amplitude sweep tests were performed in the range 0.001–100% (1 Hz, 25 °C) [23]. Moreover, frequency sweep experiments were carried out in the range of 0.1–10 Hz (25 °C), at a constant strain value within the linear viscoelastic region (LVR). The G′ values from the frequency sweep test were fitted to the following power-law equation:

(1) $$G^{\prime }=k\times \omega^{n}$$

where *k* represents the consistency coefficient (Pa∙s^n^), *ω* represents the frequency (Hz) and *n* represents the behavior index (dimensionless) [24,25].

Temperature sweep tests were carried out from 25 to 80 °C at a ramp ratio of 3 °C/min and a soak time of 120 s (1 Hz, 0.01% strain) [26].

#### 2.3.3. Statistical Analysis

The results are presented as mean values ± standard deviation of three replicates. Following the confirmation of normality and homogeneity of variances, statistical differences were evaluated using analysis of variance (ANOVA) conducted with Microsoft Excel^®^ 2019 (Microsoft Office Professional Plus, version 2506) with the support of the Real Statistics add-in. To identify significant differences, Tukey’s honest significant difference (HSD) test was applied, with the significance level set at 5% (*p* < 0.05). Principal component analysis (PCA) was conducted to evaluate similarities and differences between oleogels (Minitab Statistical Software, version 21.2, Minitab LLC, State College, PA, USA).

## 3. Results and Discussion

### 3.1. Development and Appearance of Emulsion-Templated Oleogels

**Figure 1:**
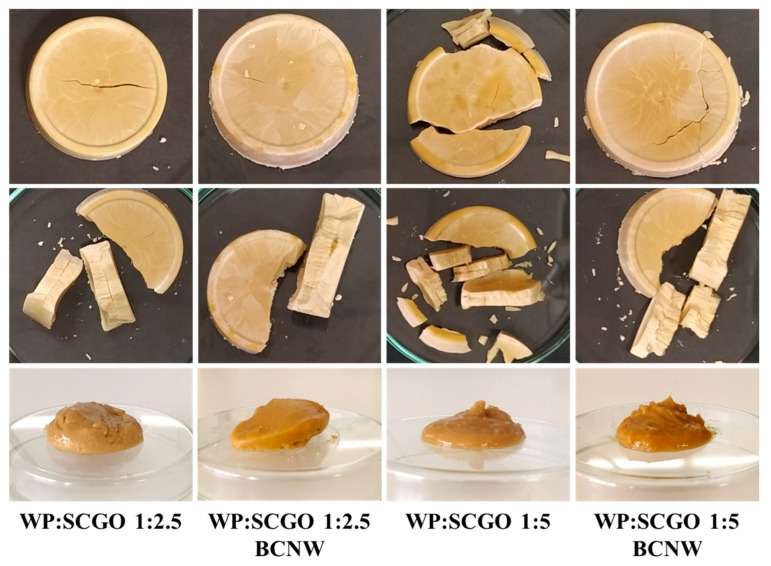
Appearance of emulsion-templated oleogels prepared with whey protein and spent coffee grounds oil (WP:SCGO) at different ratios (1:2.5 and 1:5), with and without the addition of bacterial cellulose nanowhiskers (BCNW).

The appearance of lyophilized oleogels is shown in Figure 1. Macroscopically, no noticeable differences were observed among the lyophilized forms of oleogels. After shearing, all oleogels exhibited a solid-like appearance. However, differences became more evident after shearing, as oleogels with a 1:5 WP:SCGO ratio presented a softer texture and higher flow behavior compared to those with a 1:2.5 WP:SCGO ratio. To further elucidate these differences, rheological analyses were performed and are presented in the following Section 3.2.

The color properties of the oleogels are presented in Table 1. The primary factor influencing the color of the oleogels was the inherent brown hue of the SCGO, as reflected in both Figure 1 and Table 1. The results indicated that at a 1:2.5 WP:SCGO ratio the L* parameter increased, indicating that the oleogel was lighter in color compared to the oleogel with the 1:5 WP:SCGO ratio. However, statistical analyses showed that these differences were not significant (*p* > 0.05).

### 3.2. Amplitude Sweep Tests of Emulsion-Templated Oleogels

**Figure 2:**
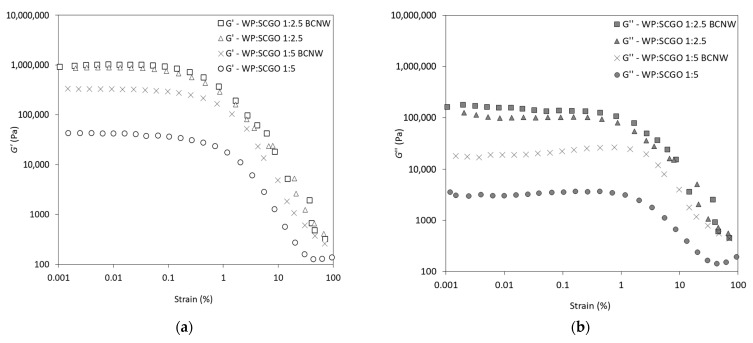
Amplitude sweep tests: (**a**) storage modulus (G′) and (**b**) loss modulus (G″) of emulsion-templated oleogels prepared with whey protein and spent coffee grounds oil (WP:SCGO) at different ratios (1:2.5 and 1:5), with and without the addition of bacterial cellulose nanowhiskers (BCNW).

As presented in Figure 2, amplitude sweep experiments were conducted to characterize the viscoelastic properties of the oleogels. The results showed that all samples demonstrated higher G′ than G″ values. The use of lower oil content in the oleogels, resulted in higher G′ values (Table 2). In particular, the WP:SCGO 1:2.5 oleogels showed significantly higher G′ values (*p* < 0.05) compared to the WP:SCGO 1:5 oleogels, indicating the development of a stronger and more elastic gel structure. This could be attributed to the higher proportion of WP, which strengthened the gel network as a result of the interparticle interactions (protein–protein and/or protein–lipid) [4,27,28]. In addition to that, the level of oil content in oleogels has a significant effect on the rheological properties. For instance, lower oil content in oleogels structured with cellulose cryogel particles contributed to higher G′ values [29], which is in line with the findings of the present study.

BCNW had a more pronounced effect on the gel strength of the WP:SCGO 1:5 oleogel, compared to the 1:2.5 ratio formulation (Table 2). This could be attributed to the stronger structuring effect of the higher WP content, which likely overlapped the effect of BCNW. Consequently, the reinforcing effect of BCNW addition on the oleogel was evident only at lower WP content. BCNW has been reported to act as a reinforcing filler in protein-based edible films [16], but this effect has not yet been investigated in oleogels. A similar reinforcing effect has been observed for palm-derived cellulose nanocrystals in a hydrogel/oleogel bigel system composed of guar gum, sesame oil, candelilla wax [30], as well as for carboxymethylcellulose (CMC) in soy protein aerogel-templated oleogels [27]. Specifically, in soy protein aerogel-templated oleogels, a dual reinforcing effect was achieved by the chemical crosslinking between citric acid and cellulose, which resulted in enhanced elasticity and mechanical strength of the oleogel [27].

Polysaccharides are frequently incorporated into protein gel matrices, but their effect on gel strength can vary depending on their nature. For example, soybean protein–κ-carrageenan oleogels exhibited a decrease in G′ [31], whereas gelatin–flaxseed gum oleogels maintained their G′ values regardless of polysaccharide addition [32]. However, several other studies, including the present one, have indicated that emulsion-templated cellulose-rich oleogels showed high gel strength [33]. This phenomenon is primarily attributed to the ability of cellulose nanofibrils to form a robust nanocellulose-matrix interface, which enables efficient stress transfer from the continuous matrix to the reinforcing phase [17]. This stress transfer reduces localized deformation in the weaker matrix and shifts the mechanical load to the stiffer reinforcing phase, thereby improving the overall strength and structural integrity of the material. In addition, a key factor contributing to this reinforcing effect is the high aspect ratio (length-to-diameter) of cellulose nanofibrils, which promotes extensive interfacial interactions and effective bridging between the cellulose nanofibrils and the surrounding matrix [34,35]. Moreover, bacterial cellulose nanofibrils can interact with the whey protein backbone through hydrophobic or hydrogen bonding, forming a complex matrix that is influenced by the ratio of these two polymers. For instance, at a high protein concentration (30%), the addition of bacterial cellulose nanofibrils resulted in distinguished interfacial viscosity changes at low (1–2%) or high concentration (4–16%) [36]. These observations are consistent with our previous findings, which demonstrated that grafting BCNW, with an aspect ratio of 20, into a whey protein (WP) matrix for edible films resulted in a significant reinforcing effect, as evidenced by the enhanced mechanical properties of the resulting WP/BCNW films [16].

All oleogels exhibited an LVR, the extension of which was influenced by the oil content. This was obvious by the G′_LVR_ (G′ at the end of LVR), which significantly increased (*p* < 0.05) as the oil content decreased. In particular, G′_LVR_ was (in 10^3^ Pa): 37.96, 315.21, 840.40 and 940.14 for WP:SCGO 1:5, WP:SCGO 1:5 BCNW, WP:SCGO 1:2.5 and WP:SCGO 1:2.5 BCNW, respectively. Furthermore, as strain increased, a critical threshold value (critical strain) was reached, which demonstrated the end of the LVR. The results in Table 2 showed that the WP:SCGO ratio affected the critical strain of the oleogels. In particular, the oleogels with 1:5 ratio exhibited a substantially lower LVR, as the critical strain was significantly lower than those with 1:2.5 ratio (*p* < 0.05). These results suggest that oleogels with higher protein content presented greater resistance to yielding and structural breakdown [21]. Conclusively, the higher protein content resulted in oleogels with higher critical strain values, consistent with previous studies [4].

Generally, the rheological parameters are considered important for the characterization of oleogels as fat substitutes in food products. Notably, the rheological data of this study showed that the oleogels exhibited G′ values similar to those of both soft and hard solid fats, depending on the WP:SCGO ratio. More specifically, the oleogels with a 1:5 ratio resembled soft solid fats, whereas those with a 1:2.5 ratio resembled hard solid fats [37].

### 3.3. Frequency Sweep Tests of Emulsion-Templated Oleogels

**Figure 3:**
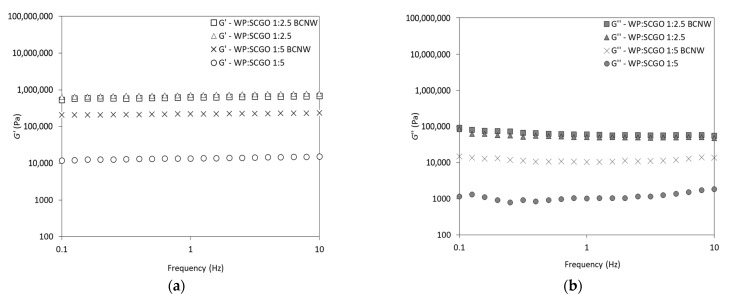
Frequency sweep tests: (**a**) storage modulus (G′) and (**b**) loss modulus (G″) of emulsion-templated oleogels prepared with whey protein and spent coffee grounds oil (WP:SCGO) at different ratios (1:2.5 and 1:5), with and without the addition of bacterial cellulose nanowhiskers (BCNW).

The viscoelastic properties of the oleogels were also determined and the results are shown in Figure 3. The G′ values were higher than G″ confirming the gel behavior of the oleogels across all frequencies. Moreover, the results align with the aforementioned amplitude sweep tests observations, concerning the patterns of G′ and G″ for the different oleogels. Specifically, oleogels with the highest WP content (2:1) presented the highest G′ and G″ values. This pattern has been observed in whey protein oleogels produced using the aerogel-templated approach [38], and recently in emulsion-templated oleogels [4]. Additionally, the incorporation of BCNW had a significant impact only when oleogels contained WP:SCGO at a ratio of 1:5. Li et al. [4] reported that higher WP content resulted in oleogels with better viscoelasticity due to a denser gel structure. Similarly, a previous study on oleogels made from soybean protein isolate and sodium carboxymethylcellulose highlighted that increased protein levels enhanced protein/polysaccharide interactions, thereby contributing to a stronger polymer structure [27].

The tan δ values at 1 Hz provide further information about the elastic nature of oleogels (Table 2). All samples exhibited sufficiently low tan δ values (<0.1), classifying them as “true” gels and indicating not only their strong gel behavior, but actually a non-fluid state [39,40]. Previous studies have shown that the protein content had a significant effect on tan δ. Specifically, hemp protein hydrogels exhibited a reduced tan δ with increasing protein content, suggesting that higher protein levels contribute to enhanced elasticity [28].

Interestingly, all oleogels appeared to be frequency-independent, which is a typical indicator of gels. Moreover, the flow behavior parameters were further confirmed by applying the Power-Law model (Table 3). The statistical analysis revealed that all oleogels exhibited similar n values, i.e., identical flow behavior indices (*p* > 0.05). On top of that, the remarkably low flow behavior indices demonstrated that G′ remained constant across different frequencies, which is characteristic of strong gel protein structures. On the other hand, k values presented significant differences among the oleogels (*p* < 0.05). More specifically, k was increased as the WP:SCGO ratio increased from 1:5 to 1:2.5. As it has been reported in the results above, the role of BCNW seemed to be insignificant at the 1:2.5 ratio (*p* > 0.05).

Clearly, preparing oleogels with higher WP content resulted in stronger gels, overlapping the effect of BCNW. However, depending on the application and the desired gel strength, this study demonstrated that the viscoelastic properties of protein oleogels can be tailored either by adjusting WP:oil ratio or by adding BCNW. The k value is an indicator of the viscoelastic nature of oleogels, and thus correlates with G′ values [28]. This is consistent with the findings of this study, where oleogels with higher G′ (Table 2) also exhibited higher k values (Table 3), and vice versa.

### 3.4. Temperature Sweep Tests of Emulsion-Templated Oleogels

**Figure 4:**
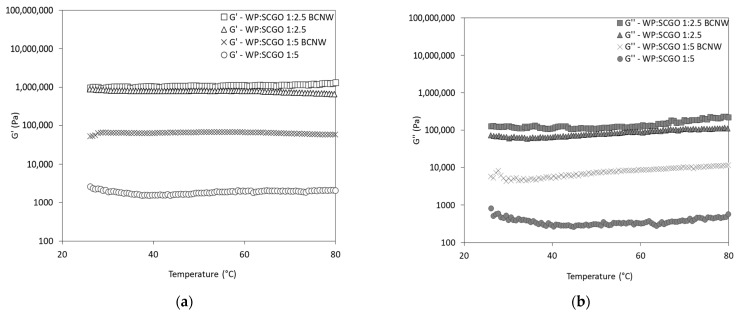
Temperature sweep tests: (**a**) storage modulus (G′) and (**b**) loss modulus (G″) of emulsion-templated oleogels prepared with whey protein and spent coffee grounds oil (WP:SCGO) at different ratios (1:2.5 and 1:5), with and without the addition of bacterial cellulose nanowhiskers (BCNW).

As shown in Figure 4, the viscoelastic properties remained stable over the tested temperature (up to 80 °C), indicating that the oleogels maintained their structure and exhibited good thermal stability. Notably, the oleogels with higher protein content (WP:SCGO 1:2.5) demonstrated higher G′ and G″ values than those with lower protein content (WP:SCGO 1:5). This suggested that the presence of more protein particles contributed to enhanced gel strength [41]. In all treatments, G′ consistently exceeded G″ and no gel-to-sol transition was observed, indicating the development of robust gel structures within the tested temperature range.

These findings are in line with recent studies investigating oleogels consisting of whey protein/sodium alginate, whey protein or soy protein/Konjac glucomannan or cellulose-rich oleogels [33,42,43].

### 3.5. Principal Component Analysis of Color and Rheological Properties of Emulsion-Templated Oleogels

**Figure 5:**
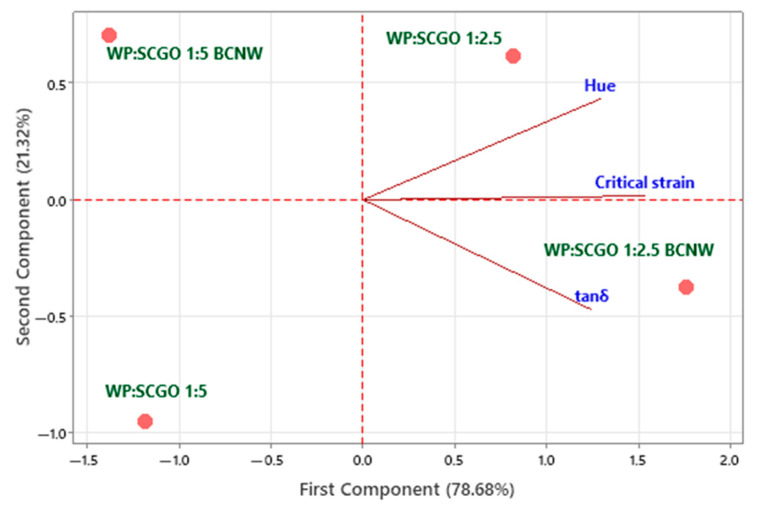
Principal component analysis for emulsion-templated oleogels prepared with whey protein and spent coffee grounds oil (WP:SCGO) at different ratios (1:2.5 and 1:5), with and without the addition of bacterial cellulose nanowhiskers (BCNW).

Three variables, tan δ, critical strain and hue, were selected to perform a PCA. Two components are shown. The first component accounts for 78.68% of the variability in the original data and the second for the remaining 21.32%. The variables were selected according to Pearson’s correlations. Color attributes that were highly correlated with hue (h°) were not included. As shown in Figure 5, the oleogels produced have been projected onto the (PC1, PC2) plane, along with vectors corresponding to the variables’ values used. Each oleogel is allocated at a different location on the plane position, which signifies its unique characteristics.

As can be observed, the WP amount is a critical factor in determining the characteristics of the oleogels. At low WP content (WP:SCGO 1:5), the oleogels are not strongly characterized by any of the selected variables. However, as WP content increases, a noticeable shift in color is observed; the oleogels present a higher hue value, indicating a lighter color. Additionally, the incorporation of BCNW leads to oleogels with a higher tan δ. While these gels remain predominantly elastic, they sustain a greater range of deformation before collapsing. As shown previously, these oleogels are structured gels with good flowability.

## 4. Conclusions

This study presented the feasibility of structuring SCGO and tailoring its rheological behavior by the use of different WP:SCGO ratios and the addition of BCNW. Oleogels with higher WP content exhibited significantly increased G′ values, indicating the formation of oleogels with greater strength and deformation resistance. Furthermore, the incorporation of BCNW had a stronger reinforcing impact on oleogels with a higher oil content (WP:SCGO 1:5 ), whereas its effect was less pronounced in those with a lower oil content (WP:SCGO 1:2.5), possibly due to the greater protein–protein interactions in the latter.

Overall, these findings highlight the potential application of the developed oleogels in a range of structured oil-based products due to their tailored rheological properties. Additionally, this study introduced an alternative approach to valorize SCGO, contributing not only to waste reduction but also to the development of sustainable food products. In this context, the development of protein-based oleogels from renewable sources aligns with the growing shift toward sustainable practices, responding to green policies that promote the production of clean-label food products.

## Figures and Tables

**Table 1:** Color parameters for emulsion-templated oleogels prepared with whey protein and spent coffee grounds oil (WP:SCGO) at different ratios (1:2.5 and 1:5), with and without the addition of bacterial cellulose nanowhiskers (BCNW).

Oleogels	L*	a*	b*	C*	*h°*
WP:SCGO 1:5	47.13 ± 1.90 ^a^	7.32 ± 0.16 ^a^	23.85 ± 1.07 ^a^	24.96 ± 0.95 ^a^	72.25 ± 2.24 ^a^
WP:SCGO 1:5 BCNW	47.98 ± 1.08 ^a^	7.47 ± 0.22 ^a^	24.94 ± 2.02 ^a^	26.03 ± 1.06 ^a^	73.32 ± 1.78 ^a^
WP:SCGO 1:2.5	52.41 ± 2.20 ^a^	7.04 ± 0.05 ^a^	25.47 ± 1.52 ^a^	26.43 ± 0.30 ^a^	74.54 ± 3.16 ^a^
WP:SCGO 1:2.5 BCNW	52.13 ± 3.03 ^a^	7.17 ± 0.50 ^a^	25.64 ± 1.30 ^a^	26.63 ± 0.95 ^a^	74.37 ± 1.93 ^a^

Means within the same column marked with different superscript letters are significantly different (*p* < 0.05).

**Table 2:** Rheological parameters of oleogels obtained from amplitude and frequency sweep tests.

Oleogels	Amplitude Sweep Tests		Frequency Sweep Tests
	G′ (×10^3^ Pa)	Critical Strain (%)	Tan δ (1 Hz)
WP:SCGO 1:5	43.45 ± 0.20 ^a^	0.040 ± 0.002 ^a^	0.074 ± 0.004 ^a^
WP:SCGO 1:5 BCNW	346.60 ± 68.30 ^b^	0.038 ± 0.001 ^a^	0.047 ± 0.001 ^b^
WP:SCGO 1:2.5	892.77 ± 24.19 ^c^	0.072 ± 0.013 ^b^	0.072 ± 0.006 ^a^
WP:SCGO 1:2.5 BCNW	977.86 ± 74.54 ^c^	0.086 ± 0.002 ^b^	0.097 ± 0.011 ^c^

Means within the same column marked with different superscript letters are significantly different (*p* < 0.05).

**Table 3:** Rheological parameters of emulsion-templated oleogels derived from frequency sweep data fitted to the power-law model.

Oleogels	*k*	*n*	R^2^
WP:SCGO 1:5	13.68 ± 0.23 ^a^	0.05 ± 0.01 ^a^	0.99
WP:SCGO 1:5 BCNW	219.21 ± 27.79 ^b^	0.03 ± 0.00 ^a^	0.99
WP:SCGO 1:2.5	718.91 ± 121.32 ^c^	0.05 ± 0.01 ^a^	0.99
WP:SCGO 1:2.5 BCNW	621.91 ± 39.54 ^c^	0.04 ± 0.01 ^a^	0.93

Means within the same column marked with different superscript letters are significantly different (*p* < 0.05).

## Data Availability

The original contributions presented in the study are included in the article, further inquiries can be directed to the corresponding author.

## Abbreviations

The following abbreviations are used in this manuscript:

BCNW	Bacterial Cellulose Nanowhiskers
LVR	Linear Viscoelastic Region
SCG	Spent Coffee Grounds
SCGO	Spent Coffee Grounds Oil
WP	Whey Protein
